# Variations of training load, monotony, and strain and dose-response relationships with maximal aerobic speed, maximal oxygen uptake, and isokinetic strength in professional soccer players

**DOI:** 10.1371/journal.pone.0225522

**Published:** 2019-12-04

**Authors:** Filipe Manuel Clemente, Cain Clark, Daniel Castillo, Hugo Sarmento, Pantelis Theodoros Nikolaidis, Thomas Rosemann, Beat Knechtle

**Affiliations:** 1 Polytechnic Institute of Viana do Castelo, School of Sport and Leisure, Melgaço, Portugal; 2 Instituto de Telecomunicações, Delegação da Covilhã, Covilhã, Portugal; 3 Faculty of Health and Life Sciences, Coventry University, CV1 5FB, Coventry, United Kingdom; 4 Faculty of Health Sciences, Universidad Isabel I, Burgos, Spain; 5 Faculty of Sport Sciences and Physical Education, University of Coimbra, Coimbra, Portugal; 6 Exercise Physiology Laboratory, Nikaia, Greece; 7 Institute of Primary Care, University of Zurich, Zurich, Switzerland; 8 Medbase St. Gallen Am Vadianplatz, St. Gallen, Switzerland; University of Rome, ITALY

## Abstract

This study aimed to identify variations in weekly training load, training monotony, and training strain across a 10-week period (during both, pre- and in-season phases); and to analyze the dose-response relationships between training markers and maximal aerobic speed (MAS), maximal oxygen uptake, and isokinetic strength. Twenty-seven professional soccer players (24.9±3.5 years old) were monitored across the 10-week period using global positioning system units. Players were also tested for maximal aerobic speed, maximal oxygen uptake, and isokinetic strength before and after 10 weeks of training. Large positive correlations were found between sum of training load and extension peak torque in the right lower limb (r = 0.57, 90%CI[0.15;0.82]) and the ratio agonist/antagonist in the right lower limb (r = 0.51, [0.06;0.78]). It was observed that loading measures fluctuated across the period of the study and that the load was meaningfully associated with changes in the fitness status of players. However, those magnitudes of correlations were small-to-large, suggesting that variations in fitness level cannot be exclusively explained by the accumulated load and loading profile.

## 1. Introduction

Quantifying training is a common practice conducted in professional sports teams, the aims of which are to determine the external and internal load imposed on players through training and to determine the acute and long-term implications of training [1,2]. Training quantification is generally conducted by using questionnaires and diaries (to control wellness status), physiological measures, serving as internal markers (e.g., heart rate responses, oxygen consumption, lactate, rate of perceived exertion [RPE], critical power, etc.), or physical measures, used as external markers (e.g., distances covered at different speeds, accelerations/decelerations, instantaneous sum of accelerations or height, number and height of jumps, etc.) [3]. The concurrence of all markers improves the overall perception of the accumulated load by players and the risk of over- or sub-optimal training stimuli [4]. However, quantifying the load does not give enough information to provide a holistic picture of the impact of training on players [5]. Indeed, a relationship between training stimuli and the development of physical fitness status may also be considered across the monitoring process; such a relationship may be defined as a dose-response relationship [6]. It seems plausible that there is a relationship between training load and the adaptations, and such process can be monitored aiming to identify is the training plan can be optimized or adjusted.

The adaptations to training stimuli and accumulated load vary in accordance to natural inter-subject variations that can be caused by multiple factors, such as age, sex, training history, psychological factors, initial training status, or the mode, duration, intensity, and frequency of training [7]. For that reason, it should not be expected that the same external load promotes similar adaptations in team sports players [8]. Nevertheless, it is well-known that the training methodology (e.g., running-based; ecological approach based on SSGs) typically used during team sports training (specifically in soccer) is highly variable, both in terms of the external, and internal, loads. This variability can be caused by the skill-based training which permits different loads to be experienced between players within the same session [9]. Therefore, such variability may be better controlled aiming to identify if some players need a complementary increase in stimuli or, in the other hand, an adjusted decrease to be in line with a proper training load.

Despite such variations, it is expected that relationships exist between accumulated training load and variations in fitness levels after the training period [10]. One of the most common and easy-to-use methods to quantify training load, and its accumulation across sessions is the RPE multiplied by the time of the training session in minutes (session-RPE). This variable serves as an indicator of the generalized load players are exposed to [11,12]. This subjective scale has been shown to have good validity and reliability levels in soccer in comparison to heart-rate-derived loads (e.g., Edwards’ methods) or to accelerometry-derived loads (e.g., players loads) [13,14].

In a study conducted during four weeks of soccer training [15], large and positive correlation coefficients (r = 0.70, 90%CI[0.30;0.89]) were observed between accumulated perceived training load (session-RPE) and percental improvements in velocity in an intermittent fitness test; indeed, these correlation coefficients were better than those obtained using Edward’s training load (r = 0.25, 90%CI[-0.28;0.67]). However, in a study conducted over nine weeks [16], the relationships between variations in an aerobic test performance (which was determined based on the velocity associated with a blood lactate of 3 mmol·l^-1^) and sum of respiratory RPE and muscular RPE, respectively, were unclear, small and negative (r = -0.17, 90%CI[-0.52;0.27]), and unclear, moderate and negative (r = -0.33, 90%CI[-0.68;0.12]), respectively. In the same study [15], large and negative associations were observed between accumulated perceived muscular load and the variations that occurred for the dominant countermovement jump (r = -0.54, 90%CI[-0.87;-0.13]) and non-dominant countermovement jump (r = -0.52, 90%CI[-0.75;-0.17]).

Though some studies, such as those mentioned above, have investigated accumulated perceived training load [15,16], no studies have tested this variable’s associations with other important indicators, such as training monotony or training strain [17]. In fact, accumulated load is only part of the overall training monitoring in team sports [18]. Variations of load within and between weeks [19], and their relationships with the load distribution can be extremely important in determining the effects of training on a player’s performance and, most of all, to understand the impact of training strategies on the adaptations of players. This knowledge could help coaches to know the training loads imposed on each microcycle and to design appropriate training tasks in order to ensure the specific soccer demands.

However, to the authors’ knowledge, no study, on the topic of dose-response, has compared markers of training monotony or strain with variations in aerobic capacity or strength in soccer players. Moreover, no study has tested the effects of accumulated training loads on the isokinetic strength of players after a period of training. Therefore, the purpose of this study was to (a) analyze the variations training load, monotony, and strain during the 10-week period; and (b) investigate the relationships between training load variables (e.g., session-RPE, training monotony and training strain) and changes in fitness and strength variables (e.g., maximal oxygen consumption (VO_2max_), maximal aerobic speed (MAS), anterior and posterior peak torques at 60°/s) in professional soccer players after 10 weeks of training.

## Methods

### 2.1. Ethical approval

The study was approved by the local ethical committee (Polytechnic Institute of Viana do Castelo, School of Sport and Leisure) with the code number IPVC-ESDL190119 and followed the ethical recommendations for the study in humans as suggested by the Declaration of Helsinki.

### 2.2. Participants

Twenty-seven professional players (age: 24.9±3.5 years old; height: 168.8±41.4 cm; body mass: 71.6±18.7 kg; fat mass percentage: 13.6±4.6%) were monitored across a 10-week period. The players were part of the same team competing in the Portuguese first league (Europe) in the season 2018/2019. Of those players, four were goalkeepers, five were external defenders, four were central defenders, eight were midfielders, four were wingers, and two were central forwards.

The study had two objectives: (a) to analyze the variations training load, monotony, and strain during the 10-week period; and (b) to determine the pre- and post-variations of fitness levels over 10 weeks and test the relationships of such fitness changes across the period with the training load variables. For objective (a), 23 players were included following the criterion of (i) not being a goalkeeper; (ii) being involved at least of 85% of the training sessions during the period; and (iii) not being injured for a period of longer than one week. For objective (b) only 14 players were included following the criterion of (i) having participated in all the assessments in the pre- and post-periods of observation; (ii) having participated in more than 85% of training sessions during the 10-week period; and (iii) not being injured for more than one week during the observed period. Players were not involved in any other training programs aside from the training regimen imposed by the coach. Players were informed before the start of the study about the design, risks, and benefits of their participation. After being informed of, and agreeing with, the terms, each player signed an informed consent.

### 2.3. Experimental design

This study followed a cohort design conducted during a 10-week period from the end of June to the beginning of September (four weeks during the pre-season and six weeks during the early competitive season). The number of training sessions and the time of the sessions can be observed in Table 1.

**Table 1:** Training sessions and time of training week during the 10-week period.

	W1	W2	W3	W4	W5	W6	W7	W8	W9	W10	Total
Training sessions (n)	6	7	7	6	6	5	5	6	5	4	57
Total time (min)	415	534	435	425	497	413	432	460	431	328	4370

W: week

Players reported their perceived effort 30 minutes after the end of each training session. These assessments were used to calculate training load. For fitness assessments, players were tested before the first training session and 48-hours after the last training session included in the 10-week period (which occurred during a week on which no matches were played).

In this study, training load variations were observed across the observed period, and the accumulated training load was correlated with the fitness variations that occurred between the pre- and post-training periods (10 weeks).

### 2.4. Training load monitoring

Foster’s 10-point scale [11] was used for player’s to report the perceived exertion approximately 30 minutes after the end of each training session responding to the question “How hard was the training session?”. In the scale, a rating of 1 corresponds to very light activity, and a rating of 10 denotes maximal exertion. The scale was applied by the same person, who had previous experience testing professional players. The players were previously informed and familiarized during a previous week with the scale to ensure answers were as accurate as possible. The scores were provided individually without the presence of other players to minimize the influence of hearing or observing the ratings given by other players, thus facilitating more precise and non-influenced individual scores. Players were allowed to mark a plus sign (interpreted as 0.5 point) alongside the integer value [20].

The score provided by each player was then multiplied by the time of training in minutes to determine session-RPE. Using session-RPE (used as measure of training load), it the following variables were calculated [17,21]: (a) the weekly training load (sum of the training loads of all training sessions during the week); (b) training monotony (mean of training load during the seven days of the week divided by the standard deviation of the training load of the seven days); and (c) training strain (sum of the training loads for all training sessions during a week multiplied by training monotony).

### 2.5. Aerobic assessment

Each player performed a test on an incremental treadmill (Technogym, Exite Run 600, Italy) test, starting at 8.0 km·h^-1^ with progressive increments of 0.5 km·h^-1^ every 30 seconds until exhaustion. The test was performed after a standardized warm-up protocol consisting of low-intensity running, to ascertain their respective maximal oxygen uptake (${\dot{\text{V}}\text{O}}_{2\text{max}}$). The players were considered exhausted whenever they volitionally declared their incapacity to continue at the predetermined pace. A treadmill slope of 2% was fixed for the duration test. Throughout the test, the participants breathed through a low dead-space (90 ml), low resistance (5.5 cm H_2_O at 510 L^.^min^-1^) facemask and turbine assembly. Gases were drawn continuously from the facemask through a 2 m sampling line (0.5 mm internal diameter) to a breath-by-breath gas analyzer (Fitmate Pro, Cosmed, Italy), where they were analyzed for O_2_ and CO_2_ (with a 200ms delay). Expired volumes were determined using a turbine volume transducer (Interface Associates, Alifovieja, US). The breath-by-breath gas analyzer was calibrated before each test using gas mixtures (Linde Gas, London, UK) of known concentrations. The turbine was calibrated before each test using a 3 L calibration syringe (Hans Rudolf, Kansas, US). Oxygen uptake was calculated and displayed on a breath-by-breath basis. The volume and concentration signals were integrated by computer, following analogue to-digital conversion, with account taken of the gas transit delay through the capillary and room temperature.

Heart rate was also monitored during the incremental test using a heart rate monitor (H10, Polar, Finland) which recorded the players’ heart rate every second and was synchronized with a local system. The maximum heart rate (HR_max_) elicited during the exercise was collected for each player. The maximal aerobic speed was determined based on the maximum speed achieved during the incremental treadmill test. The pre- and post-observed period tests were conducted in the same facility, on the same hour of the same day of the week at a stable temperature of 21°C and relative humidity of 55%.

### 2.6. Isokinetic strength

The isokinetic strength test was performed in a Biodex isokinetic dynamometer (System 4 Pro, USA), after a five-minute cycloergometer (Monark LC4, Sweden) standardized warm-up. The anterior and posterior lower limb torques were gravity-corrected, and the dynamometer calibration was executed in accordance with the manufacturer’s instructions. The lower limbs were randomly assessed after verbal and visual instruction and feedback provided by the evaluator. Players made two non-recorded trials to be familiarized with the test.

After being familiarized with the test, the players were assessed over five repetitions of concentric knee extensions and flexions at 60°/sec. Players were allowed a recovery period of 10 seconds between repetitions. Isokinetic strength ratios were calculated from measurements of maximal anterior and posterior peak torques. Peak torque deficits between lower limbs were also assessed by comparing the best trials in the extension and flexion of left and right lower limbs. The following measures were used during the statistical treatment: Extension Peak Torque at 60°/s (EPT); flexion peak torque at 60°/s (FPTL); deficit at extension (DE); deficit at flexion (DF); and ratio agonist/antagonist (Rag/An). The tests occurred in a room with a controlled temperature of 21°C and relative humidity of 55% in both evaluations (pre- and post-training period).

### 2.7. Statistical procedures

The results were presented in form of text, tables, and figures, either as means with SD, means with 90% confidence interval (90% CI), or coefficient of variation (CV%) where specified. Variations in training variables were reported as percentages in comparison to the previous week. Within-group changes considering the fitness variables were assessed using t-paired tests (to obtain the value of *p*) and standardized differences of effect size (ES) with a 90% CI [22]. The interpretation of inference’s magnitudes were used as follows [23]: < 0.2 = trivial; 0.2–0.6 = small; 0.6–1.2 = moderate; 1.2–2.0 = large; 2.0–4.0 very large; and >4.0 extremely large. The correlations between the sum of training load across the period and individualized averages of monotony and strain ratios over the period and the percentage of variations of the aerobic assessments and isokinetic strength measures between the pre- and post-training period were tested with the Pearson’s product-moment correlation coefficients (r), considering the following thresholds [24]: < 0.1 = trivial; 0.1–0.3 = small; 0.3–0.5 = moderate; 0.5–0.7 = large; 0.7–0.9 = very large; and > 0.9 = nearly perfect.

## 3. Results

**Fig 1:**
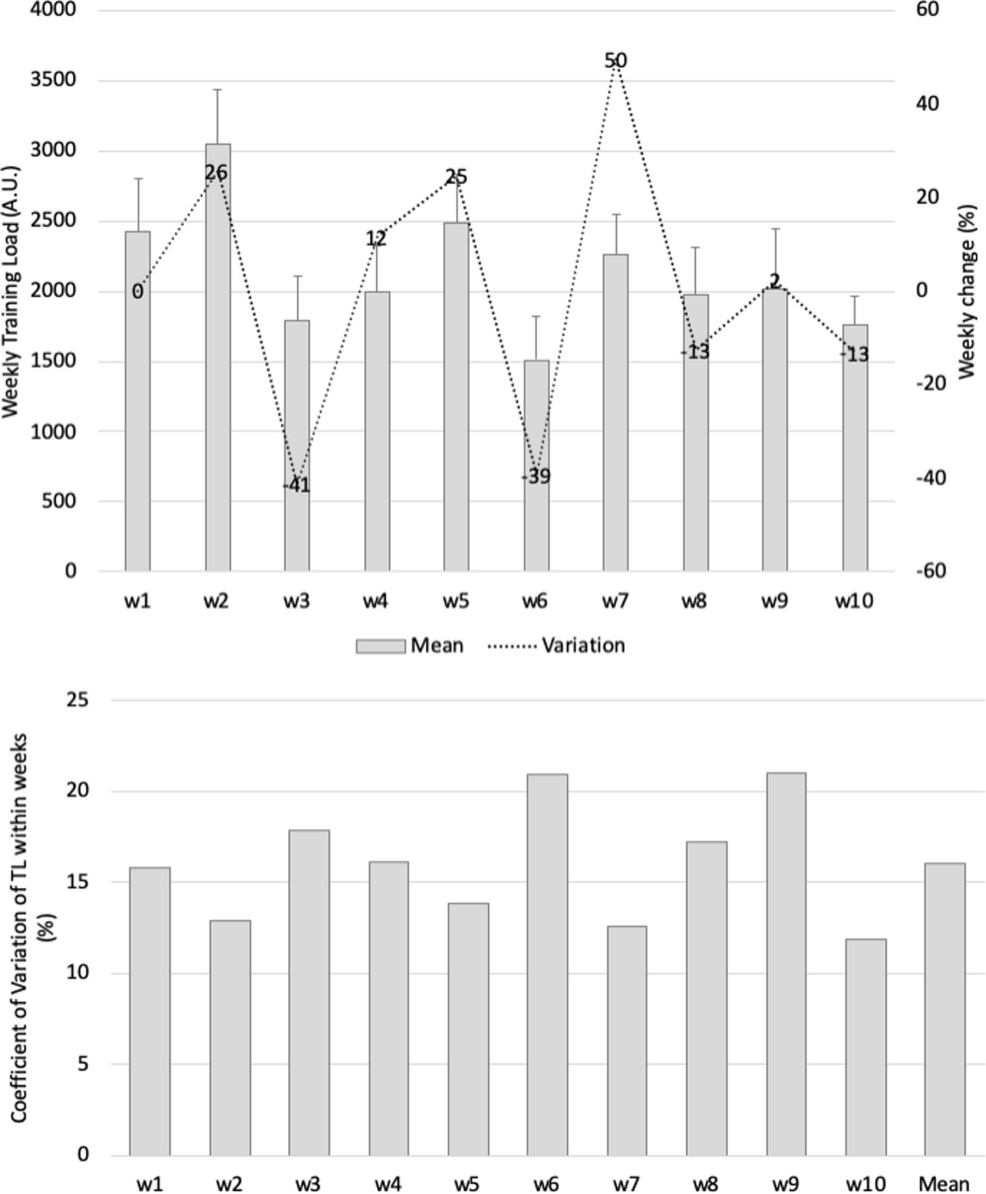
(a) Mean (SD) and weekly changes (%) in weekly training load over 10 weeks and (b) within-week load variations (CV%) and between weekly load variations (CV%).

The highest weekly training load variation reached 50% (from week 6 to week 7), and the lowest reduction (-41%) was observed from week 2 to week 3 (Fig 1). The within-week coefficient of variation was highest in week 9 (21%) and lowest in weeks 2 and 7 (13%). Across the 10-week study period, the coefficient of variation for weekly training load was 16% (mean of within week).

**Fig 2:**
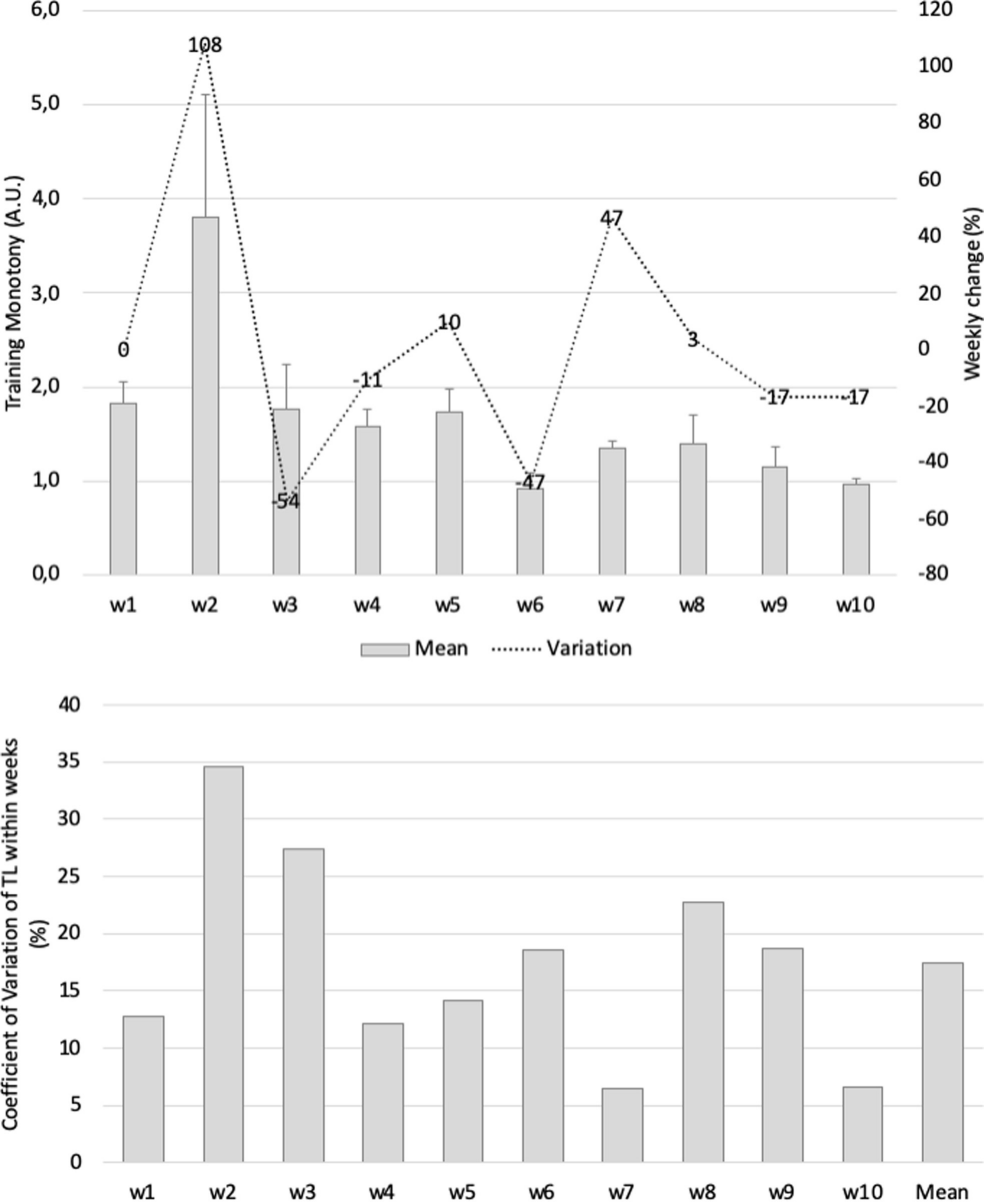
(a) Mean (SD) and weekly changes (%) in weekly training monotony during the 10-week period and (b) within-week monotony variations (CV%) and between-weekly monotony variations (CV%).

Training monotony (Fig 2) was higher in week 2 (3.8 A.U.); the highest variation occurred from week 1 to week 2 (108%). The within-week coefficient of variation (represents the average of CV of all players in each week considering all training sessions) was greatest in week 2 (35%) and lowest in week 7 (6%). Between-week training monotony variation (CV%) was 17% (mean of within week).

**Fig 3:**
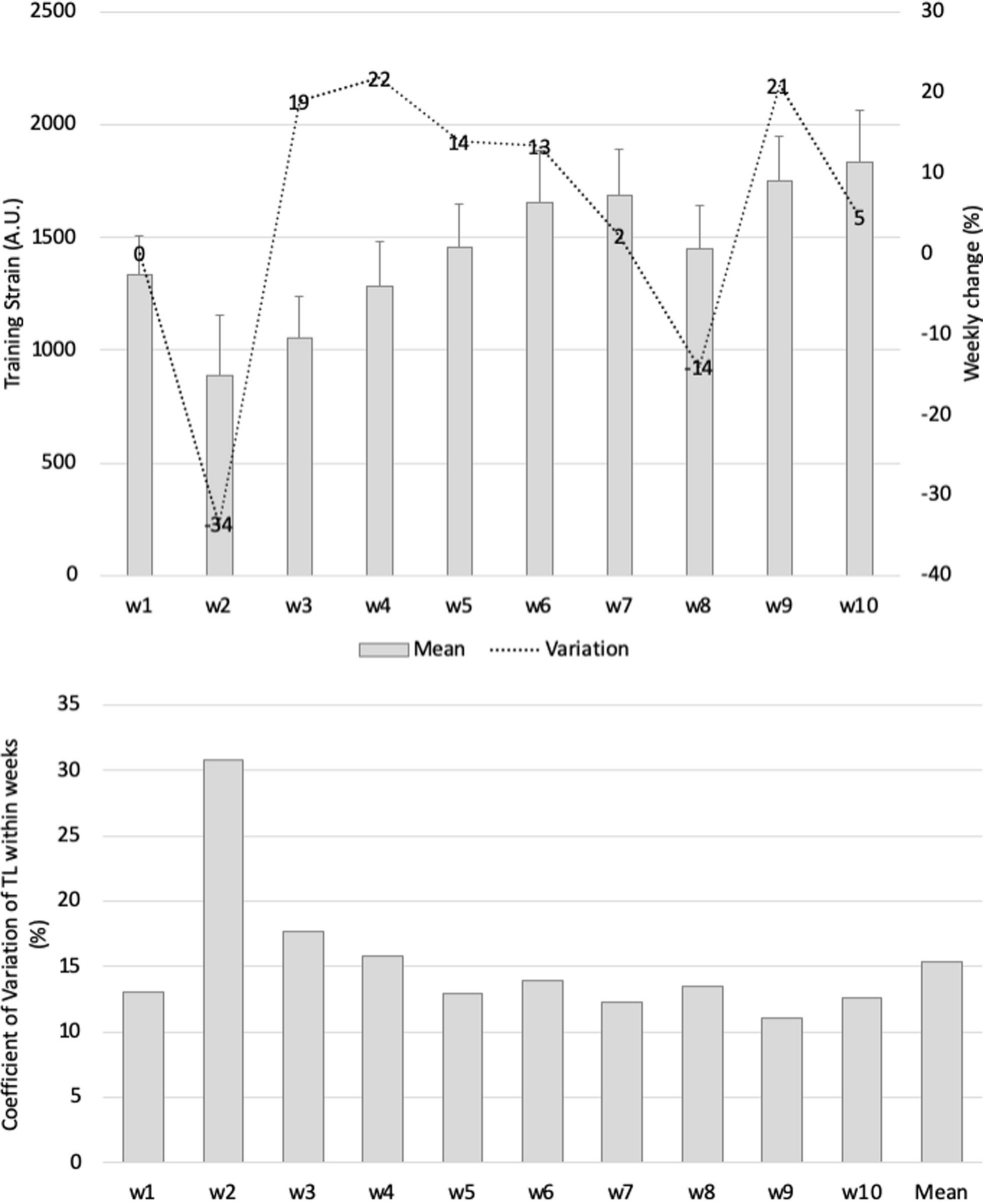
(a) Mean (SD) and weekly changes (%) in weekly training strain during the 10-week study period and (b) within-weekly training strain variations (CV%) and between-weekly training strain variations (CV%).

Training strain (Fig 3) was highest in week 9 (1751 A.U.) and lowest in week 2 (885 A.U.). The highest variation occurred from week 3 to 4 (22%). Considering the within-week variation, the greatest variation (CV%) was observed in week 2 (31%), and the lowest was recorded in week 9 (11%). The variation between weeks was 15% (mean of within week).

Within-group changes considering maximal aerobic speed, VO_2max_, and isokinetic lower limb strength can be found in Table 2. Likely small increases in VO_2max_ were found after 10 weeks of training (4.0%, [-0.1;8.1]; ES: 0.48, [-0.01;0.96]). Likely small decreases in HR_max_ were also found after the training period (-2.1%, [-4.0;-0.2]; ES: -0.41, [-0.78;-0.04]). Finally, likely small increases of peak torque during right leg flexion were observed (3.5%, [1.0;6.0]; ES: 0.32, [0.10;0.55]).

**Table 2:** Within-group differences of VO_2max_, MAS, and isokinetic strength between pre- and post-period of training. VO_2max_: maximal oxygen consumption; MAS: maximal aerobic speed; HR_max_: maximal heart rate; EPT: Extension Peak Torque at 60°/s; FPT: flexion peak torque at 60°/s; (L): left; (R): right; DE: deficit at extension; DF: deficit at flexion; Rag/An: ratio agonist/antagonist.

Variable	M(SD) Pre	M(SD) Post	% difference (Post-Pre)		*p*	Standardized difference (Post-Pre)	
			Value	[90%CI]		Value (*Magnitude*)	90%CI
VO_2max_ (ml.kg.min)	55.17(4.30)	57.34(5.09)	4.0	[-0.1;8.1]	0.09	0.48 *small*	[-0.01;0.96]
MAS (m/min)	4.76(0.31)	4.72(0.39)	-1.1	[-4.1;2.1]	0.63	-0.15 *trivial*	[-0.62;0.31]
HR_max_ (bpm)	186.50(9.14)	182.79(9.26)	-2.1	[-4.0;-0.2]	0.06	-0.41 *small*	[-0.78;-0.04]
EPT(L) (Nm)	253.31(47.78)	245.37(51.27)	-3.6	[-7.8;0.8]	0.18	-0.17 *trivial*	[-0.38;0.04]
EPT(R) (Nm)	248.56(42.58)	251.30(42.61)	1.2	[-2.9;5.5]	0.62	0.07 *trivial*	[-0.17;0.30]
DE (Nm)	12.30(11.90)	12.07(11.02)	-4.0	[-40.8;55.6]	0.74	-0.04 *trivial*	[-0.49;0.42]
FPT(L) (Nm)	154.42(22.92)	152.81(24.10)	-1.5	[-3.5;0.6]	0.36	-0.09 *trivial*	[-0.21;0.04]
FPT(R) (Nm)	161.37(15.80)	166.71(13.56)	3.5	[1.0;6.0]	0.02	0.32 *small*	[0.10;0.55]
DF (Nm)	6.48(6.61)	6.88(6.11)	12.5	[-38.0;104.0]	0.63	0.11 *trivial*	[-0.44;0.66]
RAg/An(L) (%)	62.49(15.31)	64.24(15.89)	2.6	[-0.9;6.1]	0.13	0.12 *trivial*	[-0.04;0.28]
RAg/An(R) (%)	62.04(9.54)	62.80(9.10)	0.6	[-3.8;5.1]	0.63	0.03 *trivial*	[-0.23;0.30]

VO_2max_: maximal oxygen consumption; MAS: maximal aerobic speed; HR_max_: maximal heart rate; EPT: Extension Peak Torque at 60°/s; FPT: flexion peak torque at 60°/s; (L): left; (R): right; DE: deficit at extension; DF: deficit at flexion; Rag/An: ratio agonist/antagonist

**Fig 4:**
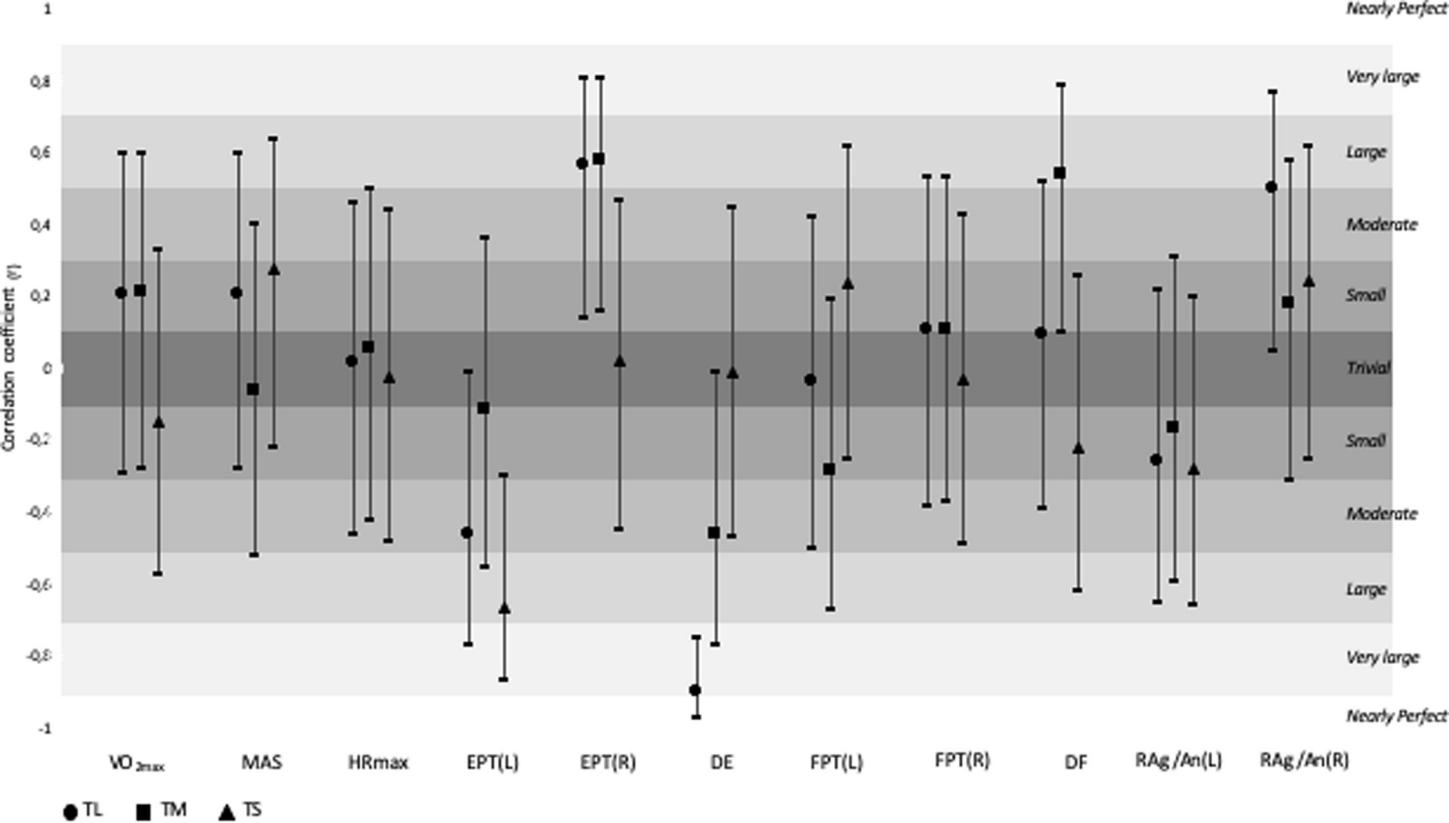
Correlation coefficients (90%CI) of sum of training load (TL), sum of training monotony (TM) and sum of training strain (TS) with % of differences (pre-post) of fitness variables.

Correlations between the sum of training variables and the percentage of variations in fitness levels were tested; the results are displayed in Fig 4. Large positive correlations were found between sum of training load and extension peak torque in the right lower limb (r = 0.57, 90%CI[0.15;0.82]) and ratio agonist/antagonist in the right lower limb (r = 0.51, 90%CI[0.06;0.78]). On the other hand, very large negative correlations were found between the sum of training load and the variations in extension deficits (r = -0.90, 90%CI[-0.96;-0.74]).

Average of training monotony was largely and positively correlated with changes in EPT(L) (r = 0.58, 90%CI[0.17; 0.82]) and deficit at flexion (r = 0.544, 90%CI[0.11;0.80]). The average of training strain was negatively and largely correlated with EPT(L) (r = -0.658, 90%CI[-0.86;-0.29]).

## 4. Discussion

Progression and controlled variations in weekly, acute training load are common in team sports, mainly because they may enhance the fitness status of players, ameliorate injury risk, and increase performance [25]. Concomitant to considering acute load, it is important to consider the stabilization of the load after the pre-season phase and the training strain over the entire period [17]. The aims of this investigation were: 1) to identify variations in weekly training load, training monotony, and training strain across a 10-week period and, 2) to analyze the dose-response relationships between training markers and aerobic assessments and isokinetic strength measures.

Regarding weekly load, it was found that the pre-season showed considerable variations in load during the first four weeks. An increase of 26% in the weekly load was found from week 1 to week 2; this was immediately followed by a decrease of 41% from week 2 to week 3. Also, weekly load stabilized during the last three weeks of the observed period. These findings are in line with those of previous studies that revealed that players tend to accumulate greater loads during the pre-season phase (namely, the first weeks) than during the in-season phase [26,27] to prepare for the increase in intensity experienced during the season [28]. Moreover, the highest coefficient of variation in the load was found in week 6 (21%), and the lowest in the last week (12%). This may suggest that as the weeks progress, the training regimen follows a more structured plan which contributes to a decrease in within-weeks variations, as avowed in previous studies [29–31].

Similar values of weekly training load were found considering similar values [16,32] and our results, for which values between 1500 and 2700 A.U. were observed in the first four weeks of the pre-season, followed by a decrease for values around 1500 A.U. during the last six weeks of training. Correspondent to weekly load decreases until stabilization, an increase in the training monotony was also found over the period of study, achieving values between 1 and 1.5 A.U. in the last five weeks.

Historically, a monotony index greater than 2 A.U. has been asserted as a risk factor for illness and overtraining in players [21]; however, such risk is only present when the load is too high, which was not the case in our study (~1500 A.U. during the period of higher monotony–week 7). The increases of training monotony in our study are not congruent (range between 0.9 and 3.8 A.U. and mean of 2 A.U.) with the results of a previous study, which used a specific periodization training method that was focused on technical-tactical ability, and reported small values (1.21–1.26 A.U.) across different phases of the season [33]. Interestingly, in our study, the training strain revealed a progressive pattern until week 7, suggesting a continuous increment during the training period.

Besides the load patterns observed in the period of this study, the dose-response relationship between loading measures and the variations in fitness levels were tested. One of the key findings in this study was that the accumulated weekly training load was largely correlated with the isokinetic peak torque during knee extension and the variation on the ratio agonist/antagonist in the right lower limb. Usually, the training process in soccer is highly dedicated to drill-based exercises characterized by reduced playing area [34], thus increasing the short-term and high-intensity actions (e.g., accelerations and decelerations) and the development of power-related football actions, thus partially explaining the large associations between training load and increases in thigh extensors [35]. On the other hand, large and negative associations were observed between accumulated weekly load and extension deficits, possibly caused by the fact that training process may reduce the deficits considering that a negative value in the case of deficit represents greater proximity to the symmetry [36].

This study was the first, to the best of our knowledge, to investigate the associations between different training load measures and crucial fitness variables in professional soccer players. Axiomatically, the present study had some limitations. The size of the sample is one of the main limitations; the fact that only players from one team were analyzed may influence the inferences made about the dose-response relationships. However, previous studies [5,16] that investigated dose-response have presented the same limitations because it is extremely difficult to monitor more than one professional team at a time. Another limitation of the present study is that no objective internal or external load measures were used. While perceived effort scales have been highly associated with heart rate measures or GPS units information [14], it would be interesting to add more information about the external load patterns to explain some of the changes in strength and MAS. Future studies should consider using larger samples and adding more objective measures to run multilinear regressions to explain the variations in determinant fitness variables.

## 5. Conclusions

Variations in weekly load revealed that the highest increases occurred from week 1 to week 2 and from 6 to 7 and that the greatest decreases occurred from week 2 to week 3 and 5 to 6, considering that this study was conducted starting on day 1 of the pre-season. Training monotony generally decreased as the weeks progressed. The training strain revealed a progressive increase during the period. Players likely increased their maximal oxygen uptake and flexion peak torque in the right lower limb and likely decreased their HR_max_ during the progressive test. The dose-response relationships revealed large and positive correlations between accumulated load and extension peak torque and ratio agonist/antagonist. However, large and negative correlations were found with deficits during extension. The results suggest that training load and the management of load should be carefully assessed to identify its influence on the progression of fitness status and deficits in isokinetic strength.

## Supporting information

S1 File Datasheet of training load. (XLSX)
