# Correction: Variations of training load, monotony, and strain and dose-response relationships with maximal aerobic speed, maximal oxygen uptake, and isokinetic strength in professional soccer players

**DOI:** 10.1371/journal.pone.0325144

**Published:** 2025-05-20

**Authors:** Filipe Manuel Clemente, Cain Clark, Daniel Castillo, Hugo Sarmento, Pantelis Theodoros Nikolaidis, Thomas Rosemann, Beat Knechtle

In the Results, the third paragraph is incorrect. The correct paragraph is: Training strain (Fig 3) was highest in week 2 (6921 A.U.) and lowest in week 9 (2038 A.U.). The highest variation occurred from week 2 to 3 (-49%). Considering the within-week variation, the greatest variation (CV%) was observed in week 2 (41%), and the lowest was recorded in week 10 (9%). The variation between weeks was 25% (mean of within week).

There are errors in Fig 3. Please view the correct Fig 3 here.

**Fig 3:**
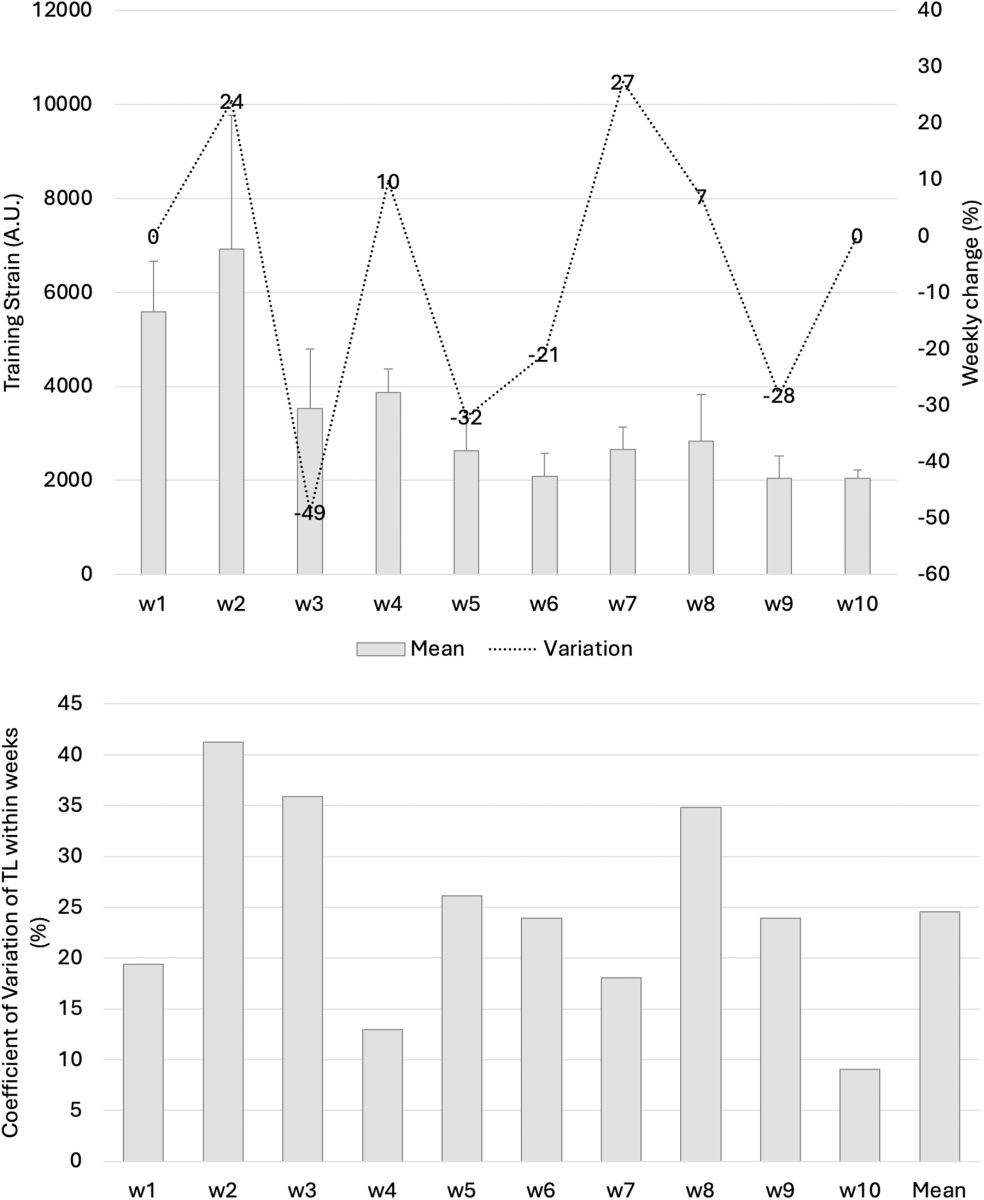
(a) Mean (SD) and weekly changes (%) in weekly training strain during the 10-week study period and (b) within-weekly training strain variations (CV%) and between-weekly training strain variations (CV%).

There are errors in S1 File. Please view the correct S1 File below.

## Supporting information

S1 File Datasheet of training load. (XLSX)

## References

[1] Clemente FM, Clark C, Castillo D, Sarmento H, Nikolaidis PT, Rosemann T, et al. Variations of training load, monotony, and strain and dose-response relationships with maximal aerobic speed, maximal oxygen uptake, and isokinetic strength in professional soccer players. PLoS One. 2019;14(12):e0225522. doi: 10.1371/journal.pone.0225522 31800617 PMC6892557

